# Alteration in kynurenine pathway metabolites in young women with autoimmune thyroiditis

**DOI:** 10.1038/s41598-024-57154-3

**Published:** 2024-03-21

**Authors:** Anna Krupa, Agnieszka Łebkowska, Marcin Kondraciuk, Karol Adam Kaminski, Irina Kowalska

**Affiliations:** 1 Department of Internal Medicine and Metabolic Diseases, Medical University of Bialystok, M. Sklodowskiej-Curie 24A, 15-276 Białystok, Poland; 2 Department of Population Medicine and Lifestyle Diseases Prevention, Medical University of Bialystok, Waszyngtona 15B, 15-269 Białystok, Poland

**Keywords:** Thyroid diseases, Translational research

## Abstract

The kynurenine pathway (KP) of tryptophan degradation includes several compounds that reveal immunomodulatory properties. The present study aimed to investigate the alteration in KP metabolites in young women with autoimmune thyroiditis (AIT) and their associations with thyroid function. The thyroid function tests, antithyroid antibodies measurement and ultrasonography of the thyroid gland have been performed in 57 young women with AIT and 38 age-matched healthy controls. The serum levels of tryptophan, kynurenine (KYN) and its metabolites were determined, and the activity of KP enzymes was calculated indirectly as product-to-substrate ratios. KP was activated and dysregulated in AIT, along with significantly elevated levels of KYN and anthranilic acid (AA), at the expense of the reduction of kynurenic acid (KYNA), which was reflected by the increase in the AA/KYNA ratio (*p* < 0.001). In univariate and multiple regression analyses, peripheral deiodinase (SPINA-GD) activity in AIT was positively associated with KYNA, AA, and quinolinic acid (QA). The merger of AA, AA/KYNA ratio, QA and SPINA-GD exhibited the highest sensitivity and specificity to predict AIT (*p* < 0.001) in receiver operating characteristic (ROC) analysis. In conclusion, the serum KYN metabolite profile is dysregulated in young women with AIT and could serve as a new predictor of AIT risk.

## Introduction

Autoimmune thyroiditis (AIT) is a prevalent thyroid disease that results in hypothyroidism. It is caused by abnormalities in autoimmune tolerance and is more common in women than in men. Approximately 0.3 to 1.5 out of every 1000 subjects per year are estimated to be affected by AIT, with a high prevalence in young women^1^. Although the cause of AIT is not fully understood, it is thought to be due to genetic factors, exposure to environmental factors, gut microbiome composition, and past infections. The concerned factors can lead to an imbalance in self-tolerance mechanisms and abnormalities in the autoimmune system^1–6^.

AIT is characterized by thyroid follicular cell atrophy, lymphocytic infiltration within the inflamed organ, and progressive fibrosis. It is generally accepted that both cellular and humoral immune responses can play a key role in AIT pathogenesis. The abnormal function of T cell subsets can lead to the breakdown of immune homeostasis in the thyroid gland, initiating the autoimmune cascade against thyroid tissue^1,3,4^. It is also believed that a functional alteration of B cells with the formation of autoantibodies is one of the first occurrences in AIT pathogenesis^7^. Consequently, the infiltration of immune cells in thyroid tissues can result in the destruction of thyroid follicular cells, leading to the development of hypothyroidism. The diagnosis of AIT typically involves the presence of thyroid peroxidase antibodies (TPOAb) and/or thyroglobulin antibodies (TgAb), along with typical ultrasound features that include decreased echogenicity^1,2,4,8^.

Iodothyronine deiodinases (DIOs) are a family of selenoproteins, which control the local and systemic availability of biologically active thyroid hormone—3,3′-5-triiodothyronine (T3) through deiodination of L-thyroxine. Three types of DIOs, whose diversified location allows for the control of thyroid hormone homeostasis in a tissue-specific manner, can be distinguished. The crucial role of DIO1 is to provide T3 for the circulatory system. DIO2 is primarily responsible for the local production of T3 inside cells, and it regulates the hypothalamus-pituitary-thyroid (HPT) negative feedback. DIO3 is considered the most important thyroid hormone-inactivating enzyme, as it generates inactive form T3 (reverse T3), lacking affinity for thyroid receptors^9,10^. The sum activity of peripheral DIOs (SPINA-GD) may be calculated in mathematical modelling created by Dietrich et al.^11^.

The kynurenine pathway (KP) of tryptophan (TRP) degradation includes several compounds and enzymes involved in numerous physiological and pathological processes. Under physiological conditions, the liver 2,3-dioxygenase (TDO) is the major contributor to TRP oxidation, whereas, in the presence of an inflammatory state, the KP is induced by activation of extrahepatic indoleamine 2,3-dioxygenase (IDO1). Kynurenine (KYN) produced as a result of these reactions is further transformed into three metabolites: 3-hydroxykynurenine (3-HKYN) by kynurenine 3-monooxygenase (KMO), anthranilic acid (AA) by kynureninase A (KYNU A) and kynurenic acid (KYNA) catalyzed by kynurenine aminotransferase (KAT). Both AA and 3-HKYN may be converted to 3-hydroxyanthranilic acid (3-HAA) through enzyme anthranilate-3-hydroxylase (A3H) or kynureninase B (KYNU B), respectively. The final compound in the KP is quinolinic acid (QA), which is formed from 3-HAA through 3-hydroxyanthranilic acid oxygenase (3-HAAO)^12–15^.

The compounds of KP play a major role in various immunological and inflammatory mechanisms. The distinct KYN metabolites can have proinflammatory, anti-inflammatory and immunosuppressive attributes as they regulate the proliferation and function of several immune cells^12,13,16^. KP is able to control the innate and adaptive immune responses, maintaining the balance between activation and inhibition of the immune system in autoimmune diseases^14^. Recent studies revealed an association between TRP metabolism and some autoimmune diseases, like multiple sclerosis^17–20^, systemic lupus erythematosus^21–23^, Sjögren's syndrome^24–27^ and psoriasis^28^. So far, scarce studies reported the alterations of KP in autoimmune endocrinopathies, like type 1 diabetes mellitus (T1DM)^29–32^ or Graves’ disease^33–35^. However, the KP has not been studied in AIT, and the potential contribution of KYN metabolites to the pathogenesis of this autoimmune disease is yet to be explored.

Our study aimed to investigate the relation of AIT with the KP in young women and to examine an association between KYN metabolites and thyroid function. Additionally, we sought to determine if KP metabolites could serve as a new predictor of disease risk.

## Results

### The characteristics of study participants

The study comprised 57 young women with AIT with a mean age of 32.45 ± 10.78 years and 38 age-matched healthy women (CON). The baseline characteristics of the study groups are presented in Table 1. No significant differences in BMI, hsCRP values and smoking status were noted between AIT and CON. Compared to CON, AIT patients had higher TPOAb and TgAb levels (both *p* < 0.0001) and FT4 concentrations (*p* < 0.01), whereas their FT3 levels, FT3/FT4 ratios, and SPINA-GD were significantly lower (*p* < 0.05, *p* < 0.01 and *p* < 0.01; respectively).

**Table 1:** Clinical and biochemical characteristics of study participants.

	Controls, n = 38	Autoimmune thyroiditis, n = 57	*p* values
Age, years	32.45 ± 10.78	32.19 ± 8.70	0.8972
BMI, kg/m^2^	24.01 ± 5.13	24.80 ± 5.87	0.5061
Current smoking, n (%)	7 (18)	7 (12)	0.4145
hsCRP, mg/L	0.55 (0.26–1.10)	0.76 (0.19–1.58)	0.7429
TgAb, IU/ml	16.36 (11.23–19.65)	165.20 (136.60–324.75)	< 0.0001
TPOAb, IU/ml	10.82 (5.00–13.22)	127.00 (52.30–257.40)	< 0.0001
TSH, µIU/ml	1.96 (1.16–2.44)	2.03 (1.39–2.97)	0.1999
FT3, pmol/l	4.75 (4.30–5.65)	4.43 (3.98–5.27)	0.0458
FT4, pmol/l	15.22 ± 1.71	16.54 ± 2.72	0.0052
FT3/FT4	0.33 ± 0.07	0.28 ± 0.06	0.0021
SPINA-GD, nmol/s	30.42 ± 6.24	26.45 ± 5.20	0.0021

Data are means ± standard deviations or medians (interquartile ranges) for continuous variables and n (%) for categorical variables.

BMI, Body mass index; hs CRP, High sensitivity C-reactive protein; TgAb, Thyroglobulin antibody; TPOAb, Thyroid peroxidase antibody; TSH, Thyroid-stimulating hormone; FT3, Free triiodothyronine; FT4, Free thyroxine; SPINA-GD, The maximum global activity of peripheral deiodinases.

### Serum kynurenine pathway (KP) metabolites and enzyme activity in the studied groups

**Figure 1:**
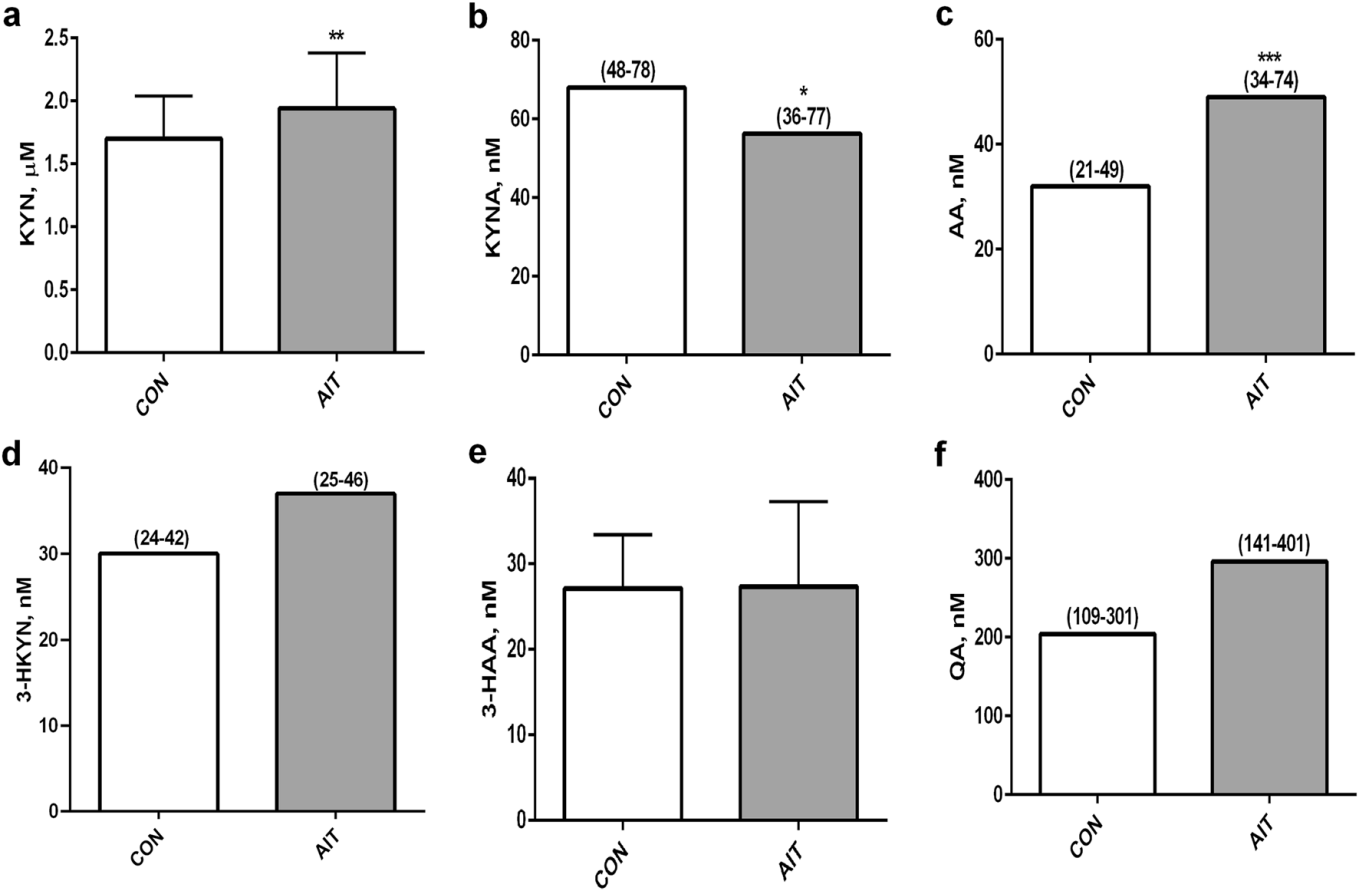
The kynurenine pathway metabolites in controls (CON) and young women with autoimmune thyroiditis (AIT), (**a**) kynurenine (KYN), (**b**) kynurenic acid (KYNA), (**c**) anthranilic acid (AA), (**d**) 3-hydroxykynurenine (3-HKYN), (**e**) 3-hydroxyanthranilic acid (3-HAA), (**f**) quinolinic acid (QA). *p < 0.05; **p < 0.01; ***p < 0.001 controls versus AIT.

There was no difference in tryptophan levels between AIT patients and CON (34.49 ± 5.82 and 34.38 ± 5.34, respectively). KYN and its further metabolites are presented in Fig. 1. Serum concentrations of KYN (Fig. 1a) and especially AA (Fig. 1c) were significantly higher in AIT women than in CON (*p* < 0.01 and *p* < 0.001; respectively). Moreover, a slightly increased level of QA, which is the final metabolite of KP, was observed in the AIT group (Fig. 1f). In contrast, KYNA level was reduced in AIT, *p* < 0.05 (Fig. 1b), whereas 3-HKYN, 3-HAA and QA concentrations in AIT patients were comparable to the control group (Fig. 1d–f).

**Figure 2:**
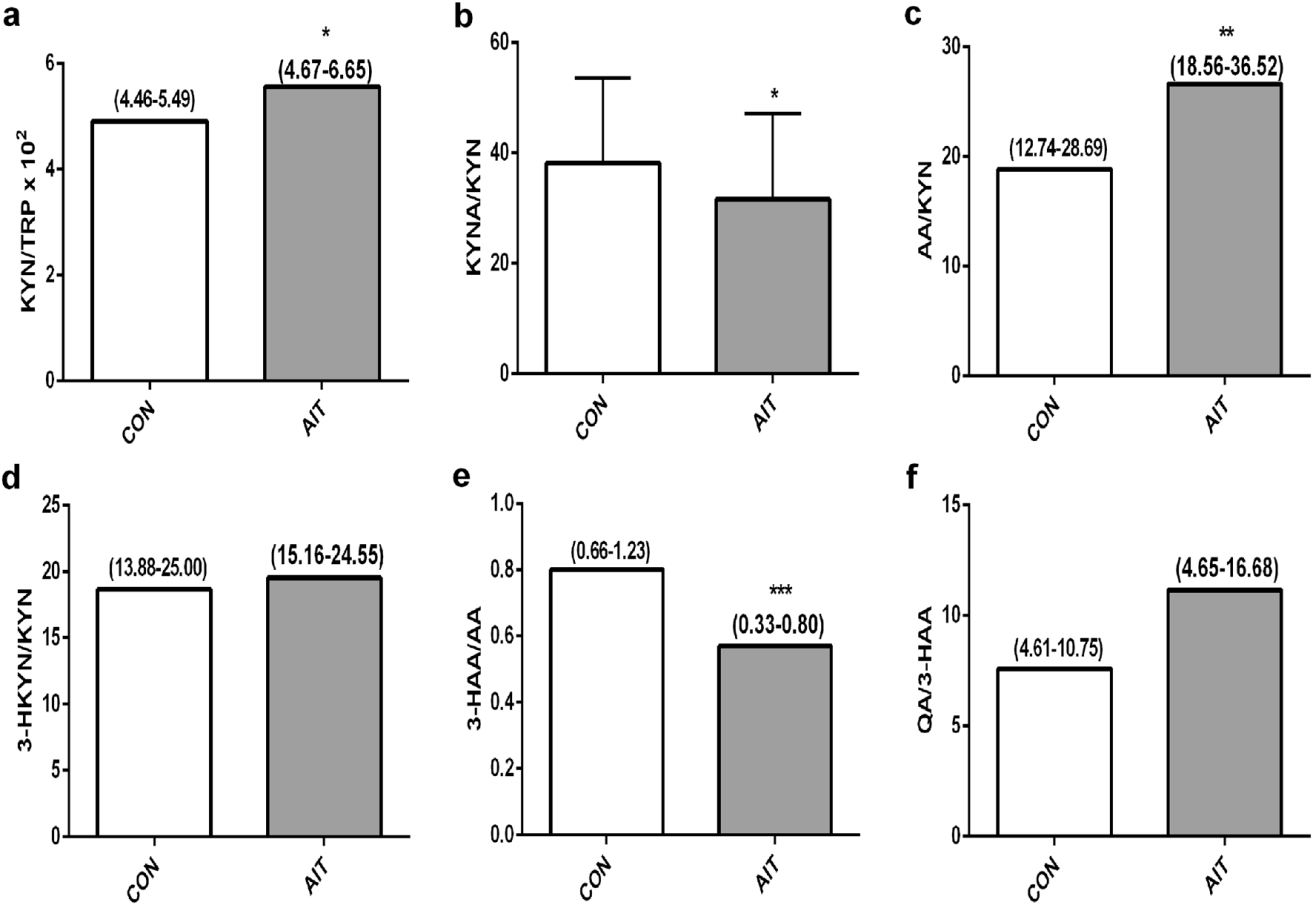
The activity of kynurenine pathway enzymes in controls (CON) and young women with autoimmune thyroiditis (AIT). The activity of individual KP enzymes were calculated indirectly, by the determination of product/substrate ratios, (**a**) IDO1, TDO activity (KYN/TRP ratio), (**b**) KAT activity (KYNA/KYN ratio), (**c**) KYNU A activity (AA/KYN ratio), (**d**) KMO activity (3-HKYN/KYN ratio), (**e**) A3H activity (3HAA/AA ratio), (**f**) 3-HAAO activity (QA/3-HAA ratio). *p < 0.05; **p < 0.01; ***p < 0.001 controls versus AIT.

The activity of KP enzymes was calculated indirectly by the determination of product-to-substrate ratios^36^. KYN/TRP ratio, a clinical index of TDO/IDO1 activity (Fig. 2a) and AA/KYN ratio, reflecting KYNU A activity (Fig. 2c) were increased in AIT compared to CON (*p* < 0.05 and *p* < 0.01; respectively), whereas KYNA/KYN ratio, reflecting KAT activity (Fig. 2b) and particularly 3HAA/AA ratio, illustrating anthranilate-3-hydroxylase activity (A3H)^12^ (Fig. 2e) were significantly reduced in the patients’ group compared to healthy women (*p* < 0.05 and *p* < 0.001; respectively). In general, AA formation from KYN was intensified at the expense of reduced KYNA production, and AA transformation to 3-HAA was attenuated, resulting in the imbalance between AA and KYNA formation (AA/KYNA ratio in controls was 0.54 (0.39–0.80), whereas in AIT it was 0.81 (0.62–1.54), *p* < 0.001). These results indicated that KP was activated and dysregulated at the AIT course.

### Association of the thyroid autoimmunity with KP metabolites in AIT and CON

**Figure 3:**
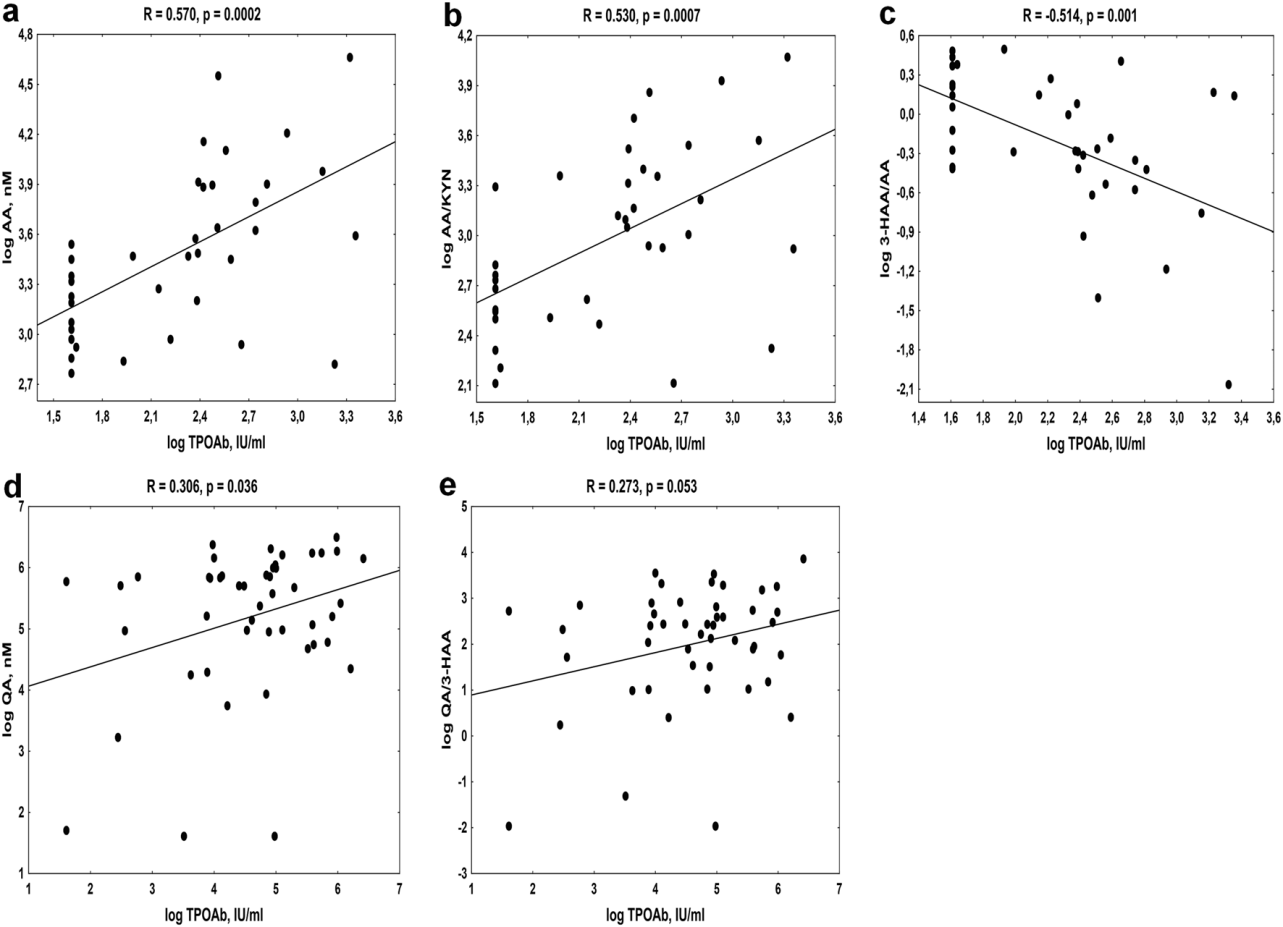
The association between thyroid peroxidase antibodies (TPOAb) levels and kynurenine pathway metabolites in controls (**a**–**c**) and young women with autoimmune thyroiditis (**d**, **e**). *AA* Anthranilic acid, *KYN* Kynurenine, *3-HAA* 3-Hydroxyanthranilic acid, *QA* Quinolinic acid.

As shown in Fig. 3, TPOAb levels were strongly and positively correlated with AA concentrations (Fig. 3a) and with the enzymatic transformation of KYN into AA (Fig. 3b) in CON. In addition, TPOAb levels were inversely associated with AA metabolism into 3-HAA in this group (Fig. 3c). On the other hand, TPOAb titer in AIT patients was associated with QA levels and with QA formation from 3-HAA (Fig. 3d,e; respectively). We also noticed the weak positive relations between TgAb and AA levels (R = 0.327, *p* = 0.048) in CON.

### Associations between KP activation and thyroid function markers in patients with AIT and CON

**Figure 4:**
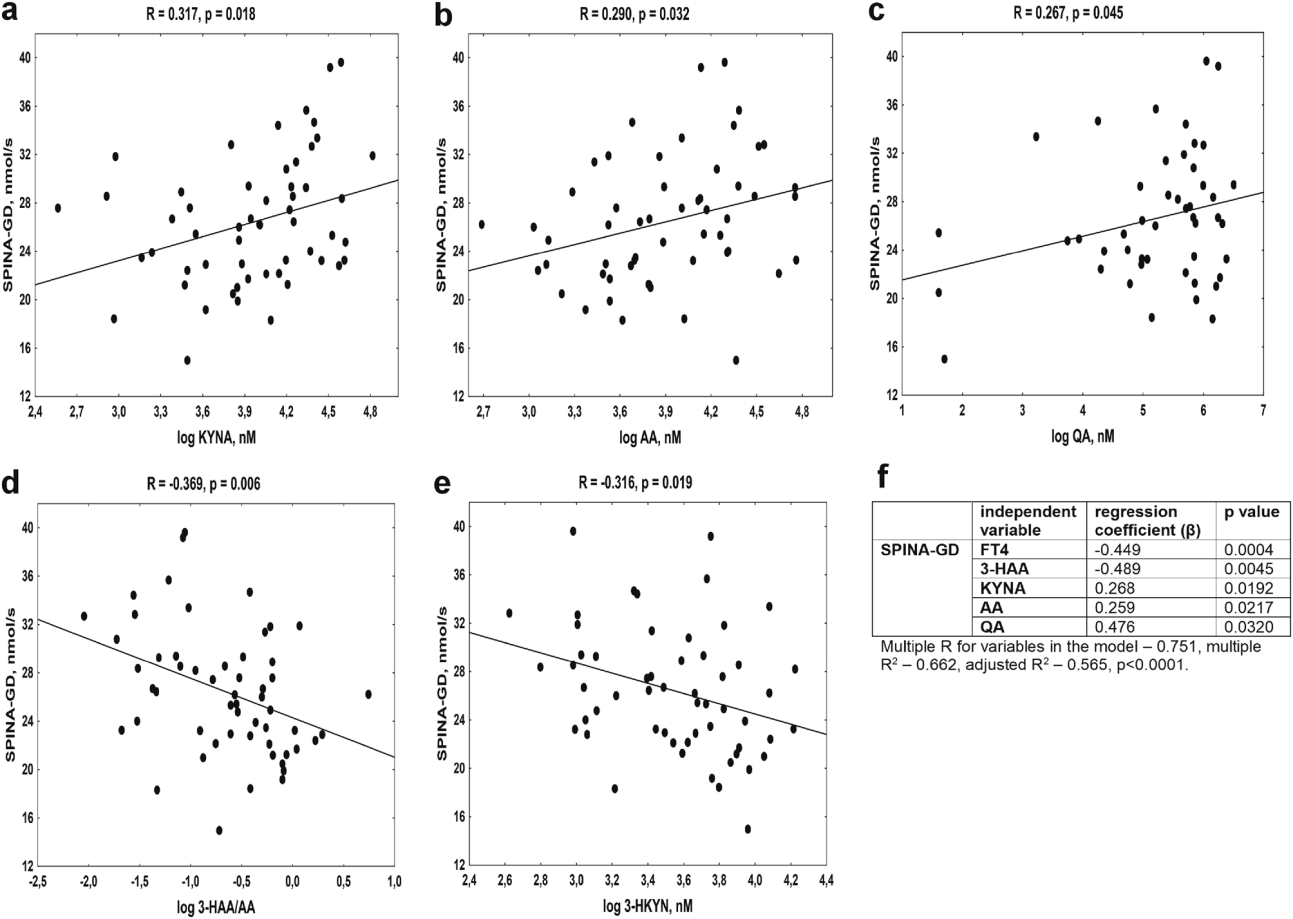
The association between the maximum global activity of peripheral deiodinases (SPINA-GD) in young women with autoimmune thyroiditis and kynurenine pathway metabolites, (**a**) kynurenic acid (KYNA), (**b**) anthranilic acid (AA), (**c**) quinolinic acid (QA), (**d**) 3-HAA/AA ratio, (**e**) 3-hydroxykynurenine (3-HKYN), (**f**) the results of multiple regression analysis with SPINA-GD as a dependent variable.

The analysis of the correlation between KP metabolites and thyroid function markers revealed the most associations between KP metabolites and SPINA-GD, as well as FT3 levels, during the AIT course. As shown in Fig. 4, SPINA-GD was positively correlated with KYNA (Fig. 4a), AA (Fig. 4b), and QA levels (Fig. 4c), whereas it was inversely associated with 3-HAA/AA ratio (Fig. 4d) and 3-HKYN (Fig. 4e). The positive relations were also noted between SPINA-GD and KYNA/KYN (R = 0.331, *p* = 0.014), AA/KYN (R = 0.287, *p* = 0.034), QA/3-HAA (R = 0.311, *p* = 0.033) and FT3 level (R = 0.661, *p* < 0.0001), while SPINA-GD was inversely related to FT4 level (R =  − 0.455, *p* = 0.004).

Based on univariate analysis, the stepwise multiple regression analysis was performed with SPINA-GD as a dependent variable. Because SPINA-GD showed a strong interrelationship with FT3, and at the same time, it determined peripheral FT3 formation, we excluded the above variable from the multivariate analysis. Stepwise multiple regression analysis confirmed that FT4 level and some KP metabolites, such as 3-HAA, KYNA, AA, and QA, were independently and significantly associated with SPINA-GD values, explaining 56.5% variability of this parameter (Fig. 4f).

**Figure 5:**
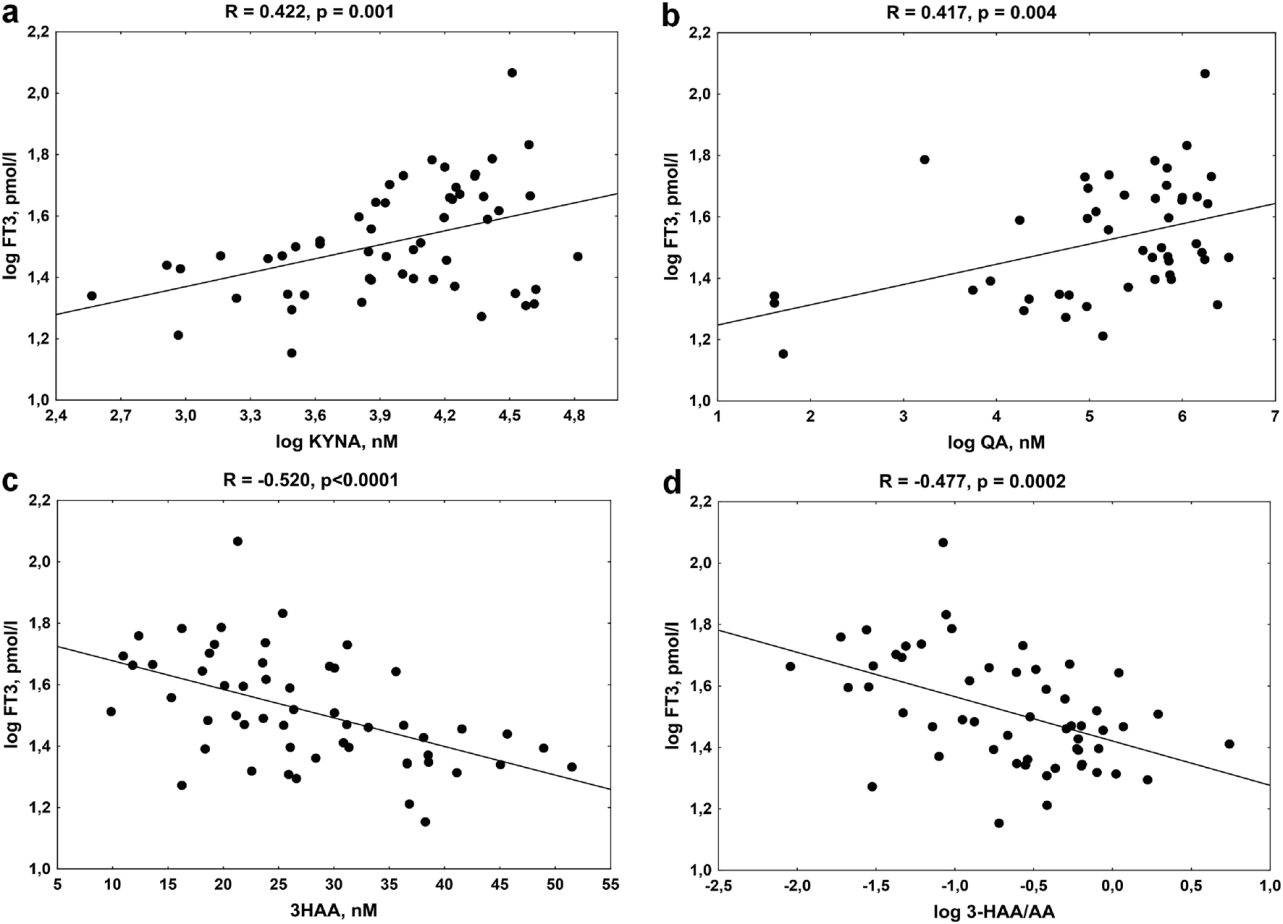
The association between the free triiodothyronine (FT3) levels in young women with autoimmune thyroiditis and kynurenine pathway metabolites, (**a**) kynurenic acid (KYNA), (**b**) quinolinic acid (QA), (**c**) 3-hydroxyanthranilic acid (3-HAA), (**d**) 3-HAA/AA ratio.

Similar relations were observed between FT3 levels and KYNA (Fig. 5a), KYNA/KYN ratio (R = 0.332, *p* = 0.013) and QA levels (Fig. 5b), whereas FT3 was inversely correlated with 3-HAA (Fig. 5c) and 3-HAA/AA ratio (Fig. 5d). We also noticed the inverse association between FT4 and 3-HAA (R =  − 0.302, *p* = 0.025) and between 3-HKYN and TSH (R =  − 0.351, *p* = 0.008).

In CON, we also found a positive relationship among SPINA-GD and KYNA (R = 0.326, *p* = 0.049), KYNA/KYN ratio (R = 0.458, *p* = 0.004), whereas FT3 level was positively correlated with AA (R = 0.340, *p* = 0.039), AA/KYN ratio (R = 0.360, *p* = 0.029), and it was inversely related to 3-HAA/AA ratio (R =  − 0.426, *p* = 0.008).

**Figure 6:**
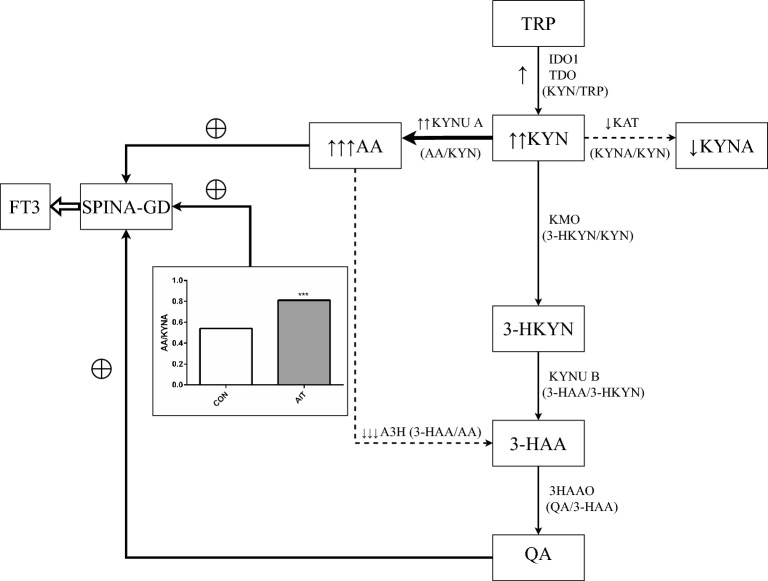
The proposed mechanism links alteration in the kynurenine pathway with disturbances in thyroid function and thyroid homeostasis in young women with autoimmune thyroiditis (AIT). During AIT, the kynurenine pathway of tryptophan metabolism is activated and alteration in this pathway occurs. The formation of AA from KYN is enhanced at the expense of KYNA generation, and at the same time there is a significant reduction in the transformation of AA into 3-HAA. The imbalance between AA and KYNA, reflected by an increase in AA/KYNA ratio and between AA and 3-HAA, resulted in AA accumulation and a slight increase in QA levels. As a consequence of this process, the activity of peripheral deiodinases (SPINA-GD) rose proportionally to AA elevation, which translated into a greater amount of biologically active form of thyroid hormone—FT3. ↑, Increase versus CON, *p* < 0.05; ↑↑, Increase versus CON, *p* < 0.01; ↑↑↑, Increase versus CON, *p* < 0.001; ↓, Decrease versus CON, *p* < 0.05; *p* < 0.01; ↓↓↓, Decrease versus CON, *p* < 0.001. TRP, Tryptophan; TDO, 2,3-Dioxygenase; IDO1, Indoleamine 2,3-dioxygenase; KYN, Kynurenine; KYNU A, Kynureninase A; AA, Anthranilic acid; KMO, Kynurenine 3, Monooxygenase; 3-HKYN, 3-Hydroxykynurenine; KAT, Kynurenine aminotransferase; KYNA, Kynurenic acid; 3-HKYN, 3-Hydroxykynurenine; A3H, Anthranilate-3-hydroxylase; 3-HAA, 3-Hydroxyanthranilic acid; KYNU B, Kynureninase A; 3HAAO, 3-Hydroxyanthranilic acid oxygenase; QA, Quinolinic acid; FT3, Free triiodothyronine.

Figure 6 schematically presents the link between the alteration in KP metabolism, SPINA-GD and the production of the active form of the thyroid hormone—FT3.

### Analysis of the predictive ability of KP metabolites

**Figure 7:**
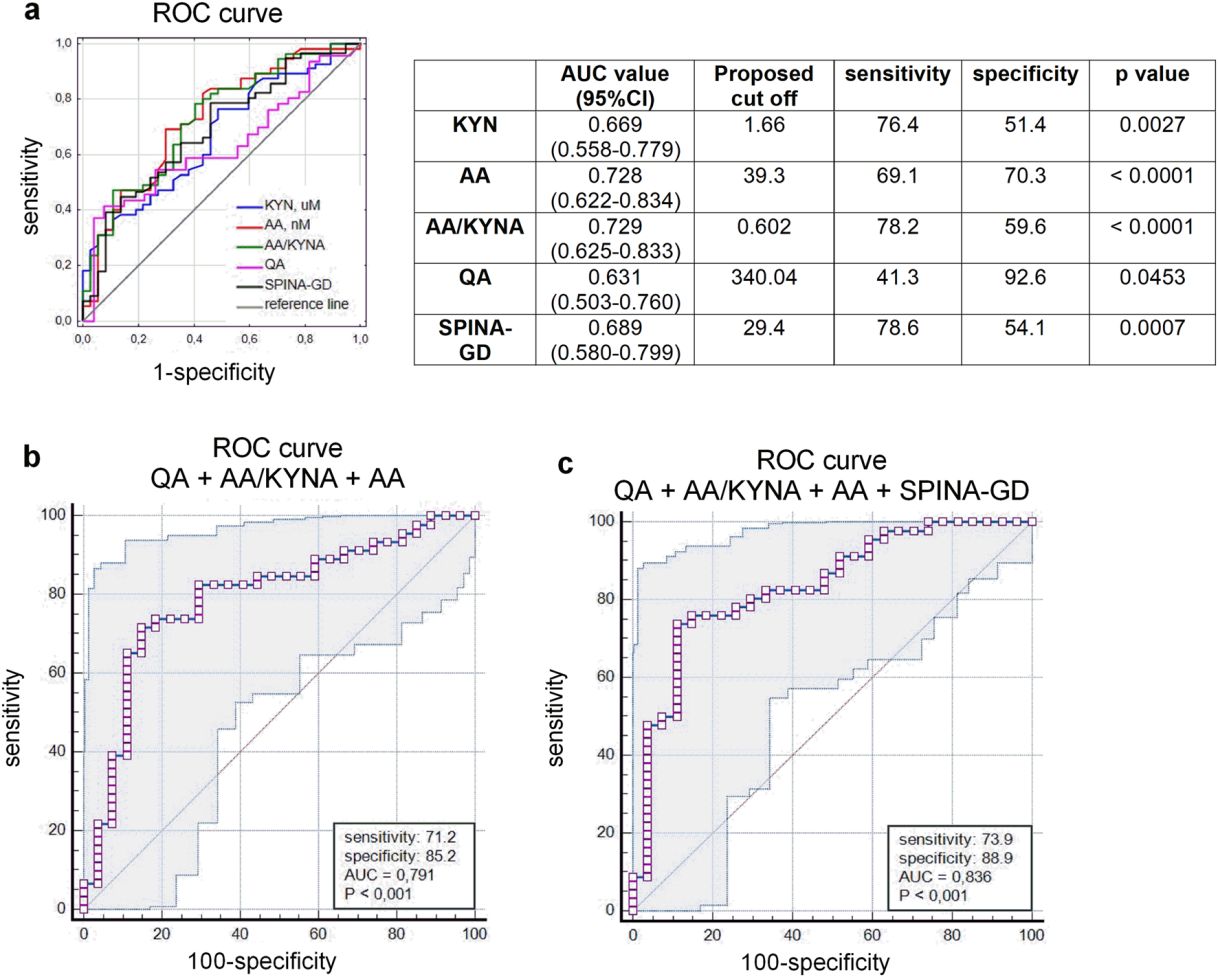
Receiver operating characteristic (ROC) curves for differentiating between AIT from control in all participants, (**a**) the predictive ability of the individual kynurenine metabolites and SPINA-GD based on ROC area under curve (AUC) values, (**b**) the predictive ability of the combination of quinolinic acid (QA), anthranilic acid (AA) and anthranilic acid to the kynurenic acid ratio (AA/KYNA) based on ROC AUC values along with sensitivity and specificity at 95% CI, (**c**) diagnostic potential of the combination of QA, AA and AA/KYNA ratio with the activity of peripheral deiodinases (SPINA-GD) by ROC analysis to distinguish AIT from controls in all participants, along with sensitivity and specificity at 95% CI.

ROC analysis was performed to examine the power of the KP metabolites to predict AIT. As shown in Fig. 7a, KYN, AA, AA/KYNA ratio, and QA achieved the statistical significance to predict AIT and the biggest AUC was observed in AA/KYNA ratio 0.729 (95% CI 0.625–0.833, *p* < 0.0001). Among the studied thyroid function biomarkers, the biggest AUC was observed in SPINA-GD—0.689 (95% CI 0.580–0.799, *p* = 0.0007) with a sensitivity of 78.6% and a specificity of 54.1%. Incorporating AA, AA/KYNA ratio and QA showed the best sensitivity and specificity to predict AIT (Fig. 7b). Additionally, the combination of these KP metabolites and SPINA-GD improved the predictive ability, yielding a ROC-AUC value of 0.836, 95% CI 0.731–0.912, with the highest sensitivity of 73.9% and specificity of 88.9% (Fig. 7c).

## Discussion

The presented study is the first attempt to investigate the involvement of the KP in the pathogenesis and development of AIT in young women. The study has revealed four significant findings: firstly, the KP's metabolism of TRP is altered during AIT, leading to an accumulation of AA and a shortage of KYNA. Secondly, a close correlation between particular KP metabolites and thyroid autoimmune status in both healthy women and AIT patients was observed. Thirdly, KP dysregulation can affect SPINA-GD and FT3 production in AIT. Finally, the combination of specific KYN metabolites and the SPINA-GD has a high diagnostic value for predicting AIT in young women.

Although dysregulation of TRP metabolism has been observed in distinct autoimmune diseases^17–28^, available literature on the activation of KP in human autoimmune endocrinopathies is limited and inconsistent, as we have previously reviewed^14^. In Graves' disease patients, the serum KYN/TRP ratio was found to be higher than in healthy individuals^33^. Nevertheless, another study discovered a lower KYN/TRP ratio and a significant increase in TRP levels in sera from Graves’ disease patients compared to matched controls^34^. Recent research by Ueland et al.^35^ confirms systemic KP activation in Graves' disease. The alteration in TRP metabolism was also noticed in type 1 diabetes mellitus (T1DM). Gürcü et al.^29^ showed that patients with T1DM had lower plasma levels of TRP and KYN than healthy individuals. Conversely, Oxenkrug et al.^30^ found that T1DM is associated with significantly elevated levels of TRP, AA, KYNA, and xanthurenic acid. While KYN concentrations did not differ between T1DM patients and controls, suggesting decreased activity of IDO1 in T1DM. In the study by Kiluk et al.^31^, serum TRP, KYN and 3-HKYN concentrations were higher, while AA levels were lower in the T1DM group in comparison to controls. Furthermore, Galderisi et al.^32^ showed higher urine levels of KYN in children with T1DM than in healthy children.

In the present study, we detected the activation of the KP and its abnormal regulation in the sera of young women with AIT compared to healthy controls. The main finding was that KYN and particularly AA levels were elevated, whereas KYNA was reduced in AIT, leading to the imbalance between AA and KYNA levels. The remaining evaluated metabolites of KYN, such as 3-HKYN, 3-HAA and QA were unchanged in the AIT group compared to controls. The above alterations in KP metabolites resulted from the dysregulated activity of KP enzymes, as shown in Fig. 2. The KYN/TRP ratio has become a widely accepted clinical marker of immune system activation, currently, it has become clear that apart from the reduced availability of TRP, likewise, downstream KYN metabolites can have a direct immunomodulatory effect^15^. The observed in this study accumulation of AA was due to intensified KYNU A activity, which was reflected by a rise in AA/KYN ratio, and reduced possibility of transformation of AA to 3-HAA by anthranilate-3-hydroxylase^12^, as has been illustrated by a significantly reduced 3-HAA/AA ratio. A similar shift in KP metabolism has been presented by Darlington et al.^37^, who described a decrease in the ratio of plasma 3-HAA/AA in a variety of neurological and diverse inflammatory disorders, including Huntingtons disease, chronic brain injury, stroke, osteoporosis and depression. The authors proposed that this decrease may either reflect an inflammatory disease or may be an anti-inflammatory response. Badawy^13^ introduced the hypothesis that the decreased 3-HAA/AA ratio may be a protective response against inflammation in clinical conditions, as both KYNA and AA possess anti-inflammatory properties, and KYNA can increase the formation of AA by activating KYNU A. However, in the present study, KYNA concentration and KYNA/KYN ratio were lower in AIT than in controls, suggesting that KYNA was rather unable to play the role in KYNU A activation. The marked and robust increase of serum AA, the elevation in AA/KYN ratio associated simultaneously with reduced KYNA and KYNA/KYN ratio observed in our AIT patients indicated that rather the substrate (KYN) deficiency in the arm KYN-KYNA was due to the shift in KP metabolism favouring AA formation, and it was responsible for the reduction of KYNA production.

Another explanation for the increased AA formation could be a deficiency in vitamin B2, based on studies conducted on experimental models^38,39^. Vitamin B2, also known as riboflavin, acts as a co-factor for KMO, an enzyme that catalyzes the formation of 3-HKYN from KYN^40^. Although in the available literature there is no data on vitamin B2 deficiency in AIT, research has shown that children with T1DM, another autoimmune disease unveiling a close genetic link with AIT^41^, have a riboflavin deficiency^42^. Therefore, such clinical conditions might be possible in AIT as well. When vitamin B2 is deficient, KYN is more accessible for AA biosynthesis, decreasing 3-HKYN formation. In animals, vitamin B2 deficiency has been linked to an increase in AA excretion and a decrease in 3-HKYN^39^. However, our results show a slight increase in 3-HKYN levels and an unchanged 3-HKYN/KYN ratio, which contrasts with previous findings. While an increase in AA and a decrease in the 3-HAA/AA ratio has been observed in other autoimmune and inflammatory diseases^30,37^, a simultaneous decrease in KYNA has not been reported. Therefore, we postulate that the AA/KYNA ratio increase may be a specific feature of AIT (Fig. 6).

Elevated TSH, TPOAb and TgAb positivity are predictors of thyroid dysfunction^1,2,4^. Elevated serum TPOAb levels are commonly acknowledged as the best serological marker of autoimmune thyroiditis, detecting in about 95% of patients with clinical features of AIT, despite they might be present in 10–15% of non-AIT patients^43^. TPOAb from AIT patients can activate the complement cascade reaction, destroy thyroid cells and act as a competitive inhibitor of enzymatic activity^44,45^. The young patients often present lower TPOAb levels, and occasionally negative results may appear in patients with clinical features of the disease^46^. TgAb, directed against thyroglobulin, are less sensitive than TPOAb (positive in about 60–80% of AIT patients), hence their lower usefulness in predicting thyroid dysfunction. The functional consequence of TgAb is unclear, as they do not cause thyroid cell destruction and could be detected in about 10–15% of healthy subjects and patients with non-thyroid immune disorders^43^. According to Brent’s hypothesis^47^, the indicated types of anti-thyroid antibodies can represent the different aspects of the autoimmune response against the thyroid gland: TgAb might reflect an innate type of immune response, and they can be present at disease onset, while TPOAb can be created in a later adaptive immune response, during an immune escalation. In the present study, we noticed that even the low status of thyroid autoimmunity, recorded in CON, was able to cause several distinct characteristic alterations in KP, namely accumulation of AA and decrease in its transformation into 3-HAA. While the status of TPOAb was associated with QA generation in the AIT group. These results, for the first time, reveal the close relationship between KP activation and thyroid autoimmune status both in physiological conditions, as well as in the AIT course. Moreover, data obtained from CON indicate that the first alteration in KP, triggered by thyroid autoimmunity in AIT, is most likely the formation and accumulation of AA, leading to a reduction in KYNA formation due to a shortage of KYN.

The clinical significance of alterations in enzymes and metabolites of KP in a spectrum of autoimmune diseases, especially in autoimmune thyroid disease, is poorly understood. In the scarce animal studies, the role of local IDO1 expression in the experimental autoimmune thyroiditis (EAT) model was investigated^48–50^. In the above mouse, so-called NOD-H2^h4^ model, autoimmune thyroiditis develops as T cell-mediated disease, resulting in the destruction of the thyroid follicles. It has been shown that the blockade of cytotoxic T lymphocyte-associated protein 4 (CTLA-4) exacerbated autoimmune thyroiditis in NOD-H2^h4^ mice and induced an expression of IDO1 in mouse thyroid glands and peripheral antigen-presenting cells^48^. While the injection of adenovirus expressing IDO1 directly into the thyroid gland of NOD-H2^h4^ mice attenuated autoimmune thyroiditis^49^. The local IDO1 expression has been interpreted as a counterregulatory mechanism, protecting the thyroid glands from autoimmune attack^48,49^. Recently, Qiu et al.^50^ showed that the administration of a herbal compound, *Prunella vulgaris*, induced IDO1 mRNA and protein expression in the spleen and intestine, increased serum KYN/TRP ratio and promoted the expansion of splenic regulatory T cells in rats with EAT. As explained by the authors, IDO1 expression was a counterregulatory mechanism, by which animals with EAT tried to reduce the self-activated immune response at the beginning of the disease.

In the present study, we tried to establish the potential significance of KP activation in thyroid function in the AIT course. The most associations were detected between KP metabolites and SPINA-GD, as well as FT3 levels in this group. Comparable relations were also present in healthy women. SPINA-GD estimates the maximum global activity of peripheral deiodinases per unit of time^11^, whereas FT3 is converted from FT4 by deiodinases in peripheral tissues. Therefore, the amount of biologically active form of thyroid hormone is highly dependent on SPINA-GD. The observed effect of KP on thyroid parameters varied depending on the KYN metabolite tested, namely KYNA, AA and QA positive impact on SPINA-GD, whereas 3-HAA formation and 3-HKYN had the opposite effect. The results of the univariate analysis were confirmed in the stepwise multiple regression analysis (Fig. 4), explaining about 57% variability of SPINA-GD in women with AIT. The indicated results suggest that AA accumulation and QA generation, coincidentally with simultaneously reduced transformation of AA into 3-HAA, can play an important role in the maintenance of the appropriate function of peripheral deiodinases and FT3 generation, which both are impaired at the course of AIT. This finding could be considered as a compensatory mechanism, counteracting the deficit of active thyroid hormone in the course of AIT. Based on the above results, we postulated the working hypothesis, linking the alteration in KP metabolism with disturbances of thyroid function during the AIT course (Fig. 6).

Nowadays, KP is actively studied as a prognostic biomarker in patients with inflammatory and autoimmune diseases. Lim et al.^18^ showed that KP metabolites in the serum of patients with multiple sclerosis may have applications as disease biomarkers. The increased levels of KYN and KYNA have been postulated as novel diagnostic biomarkers for Kawasaki disease in children^51^. Park et al.^24^ suggested that serum KYN/TRP ratio can be a potential biomarker of fatigue in the primary Sjögren's syndrome. In the study of Silva et al.^52^ KYN has been acknowledged as one of the predictors of chronic kidney disease. In the current study, ROC curve analysis revealed a high diagnostic value of several serum KP metabolites (AA, QA and AA/KYNA ratio) in AIT prediction. We also found that the combination of these parameters with SPINA-GD was the best marker to discriminate healthy from diseased young women (Fig. 7). During the AIT course, diverse thyroid functional states might exist according to varied degrees of thyroid destruction. As a result, most patients may not experience specific symptoms and are therefore not diagnosed with AIT^3^. To summarize, the results of the present study indicate that several KP metabolites, particularly in combination with SPINA-GD, could be used as the potential predictive markers of autoimmune thyroiditis in young women.

Our study has limitations that must be acknowledged. Firstly, as a cross-sectional study, we could not establish a causal relationship between the changes in KYN metabolites and thyroid function/thyroid homeostasis parameters in the AIT course. Secondly, despite the study being statistically powered and having a population that exceeded requirements, the sample size was relatively small. Further studies with a larger number of patients would be necessary to confirm the results. Additionally, since the study only included young women, the uncertainty of whether older women and males with AIT would exhibit similar changes in KYN metabolism requires further investigation. However, our study offers a new perspective by suggesting that the serum KYN metabolite profile can be considered a new sensitive biomarker for predicting AIT in young women.

In conclusion, the present study proved that serum KP metabolites were altered in the AIT course in young women. The accumulation of AA at the expense of KYNA was observed, and the aforementioned disturbances in KP were associated with thyroid autoimmune status, as well as with thyroid function markers. ROC curve analysis revealed that several of the KP metabolites, such as combined AA, AA/KYNA ratio and QA, could serve as a new predictor of AIT risk, and the addition of SPINA-GD to these compounds was the best marker for distinguishing healthy from the diseased women. Our findings underscore the value of continued research in the area and suggest that targeted KP metabolites may represent a promising avenue for future investigation. However, due to the cross-sectional design of the above research, it remains unclear whether the abnormal metabolism of kynurenine contributes directly to the pathogenesis of AIT or is solely a biomarker of the disease.

## Methods

### Study group

Participants of the current study were selected among young women (aged between 19 and 50 years old) residents of Bialystok, who were invited to voluntarily participate in the study. The recruitment lasted between March 2021 and October 2022 in the Department of Internal Medicine and Metabolic Diseases Medical University of Bialystok. In all participants, medical history data were collected by completing a questionnaire containing information about a history of thyroid disease, patients’ medications, levothyroxine substitution, hormonal contraception and the presence of other chronic diseases. The weight and height measurement was performed on a body composition analyzer InBody 570 (InBody Co., Eschborn, Germany) and body mass index (BMI) was calculated as weight (in kilograms) divided by height (in meters) squared. In all participants, the ultrasound examination of the thyroid gland was performed by the ultrasound system (USG APLIO 300 type TUS-A300, Toshiba, Japon) with a linear-array transducer (sonda PUT-375BT). All participants were examined by a well-trained ultrasound physician, who was blinded to laboratory results.

AIT diagnosis had been made by clinical examination of the thyroid gland, elevated TPOAb and/or TgAb titers and characteristic ultrasound features of autoimmune thyroid disease observed in conducted ultrasonography^1,2,4,8^. The exclusion criteria were as follows: the presence of diabetes mellitus and other autoimmune or endocrine disorders, hepatic or renal failure, other chronic diseases, pregnancy/lactation, hormonal contraception or any chronic pharmacotherapy, except levothyroxine substitution.

The healthy controls were defined based on the thyroid tests (thyroid-stimulating hormone (TSH), free triiodothyronine (FT3) and free thyroxine (FT4) within the normal range), the absence of TPOAb or TgAb and the regular image of the thyroid gland in the ultrasound examination. The exclusion criteria for the control group were the same as for AIT patients.

A total of 95 participants met the above criteria—57 women with AIT and 38 age-matched healthy women (CON). Thirty-two patients (56%) in the AIT group were treated with thyroid hormone replacement therapy (mean levothyroxine dosage of 1.14 ± 0.41 µg per kilogram of body weight per day).

### Ethical approval

The study complied with the principles of the Declaration of Helsinki, and the protocol was approved by the Ethics Committee of the Medical University of Bialystok, Poland (approval no. APK.002.404.2020). All enrolled subjects provided written informed consent for their data to be used in this study.

### Laboratory assays

To ensure accurate readings, the samples of venous blood were collected on the day of the clinical examination in the morning between 8:00 and 10:00 a.m. The blood was drawn from the antecubital vein after at least 12 h of fasting. After collection, the serum was separated through centrifugation at 3000 rpm for 10 min at 4 °C and stored at − 80 °C for assessment of biochemical parameters and KP metabolites.

Serum TSH, FT4, FT3, TPOAb and TgAb values were measured by electrochemiluminescence assays (ECLIA) on ROCHE Cobas E411, Switzerland. The reference ranges for TSH, FT3 and FT4 were as follows: 0.27–4.20 µIU/ml, 3.1–6.8 pmol/l and 12–22 pmol/l, respectively. Positivity for TPOAb or TgAb was diagnosed as the values were > 34 IU/ml or > 115 IU/ml, respectively. High sensitivity C reactive protein (hsCRP) was measured by particle-enhanced immunoturbidimetric assay on ROCHE Cobas C303, Switzerland, with the reference value < 5 mg/L.

### Calculated parameters of thyroid function

We used the FT3 to FT4 ratio (FT3/FT4) as a simple estimate of the conversion of thyroxine to triiodothyronine. In addition to this crude ratio, we used the online freely available SPINA Thyr 4.2 for Windows software for the calculation of the SPINA-GD. The parameter was calculated based on levels of TSH, FT4 and FT3, as was previously described by Dietrich et al.^11^. SPINA-GD estimates the maximum global activity of peripheral deiodinases per unit of time and shows a linear relationship with the T3/T4 ratio in euthyroid patients. The reference range for SPINA-GD is typically 20–60 nmol/s^11^.

### Determination of kynurenine pathway metabolites

Tryptophan and KP metabolites were determined by high-performance liquid chromatography (HPLC). The chromatographic equipment was an Agilent Technologies 1260 series LC system composed of G1321 binary pump VL, G1379B degasser, G1329A autosampler, G1330B thermostat for autosampler, G1316A column thermostat, G1315C diode array, G7121B fluorescence and Hewlett Packard HP1046A electrochemical detectors.

Deproteinized samples were prepared by adding 25 μl 2 M perchloric acid into the 100 μl of serum. The acidified samples were vortexed, kept at 4 °C for 2 min, and then centrifuged for 30 min at 14,000 rpm at 4 °C. 2 μl of the supernatant was injected into the HPLC system for analysis. Kynurenine (KYN) concentration was measured using the Reprospher 100 C18 3.5 μm 2 × 150 mm column. The effluent was monitored with a diode array detector (KYN-365 nm, TRP-280 nm). The mobile phase was composed of 0.1 M acetic acid and 0.1 M ammonium acetate (pH 4.6) containing 8% of acetonitrile and it was pumped at a flow-rate of 0.18 ml/min. Chromatography was carried out at 24 °C.

3-hydroxykynurenine (3-HKYN), was measured using an electrochemical technique. The potential of the working electrode of the electrochemical detector was 0.6 V. The mobile phase consisted of 0.1 M triethylamine, 0.1 M phosphoric acid, 0.3 mM EDTA, 8.2 mM heptane-1-sulfonic acid sodium salt, containing 8% of acetonitrile and was pumped at a flow-rate of 0.3 ml/min, 5 μl of the supernatant was injected into HPLC system for analysis. The prepared sample was separated on the Waters column (Spherisorb 3 μm ODS 2 2.1 × 150 mm). Chromatography was carried out at 24 °C.

Kynurenic acid (KYNA), anthranilic acid (AA) and 3-hydroxyanthranilic acid (3-HAA) concentrations were determined using the Phenomenex PEPTIDE 3.6 μm XB-C18 4.6 × 250 mm column. The effluent was monitored by using a programmable fluorescence detector. Excitation and emission wavelengths were set at 254/404 nm for KYNA, AA and 3-HAA. The mobile phase consisted of 100 mM zinc acetate, and 45 mM acetic acid, containing 16% acetonitrile was pumped at a flow-rate of 0.5 ml/min, 1 μl of the supernatant was injected into the HPLC system for analysis. The output of the detector was connected to a single instrument LC ChemStation. Chromatography was carried out at 24 °C.

Quinolinic acid (QA) concentrations were measured by enzyme-linked immunosorbent assay (ELISA) using a commercially available kit Immusmol from Immusmol SAS, Bordeaux, France. QA detected by this kit shows a high degree of correlation (R^2^ = 0.995) with the determination of this substance using liquid chromatography with tandem mass spectrometry (LC–MS/MS), as has been proved by the kit manufacturer.

### Statistical analysis

The sample size calculation showed that 30 subjects in each group were required to obtain a statistical power of 80% with a two-tailed type I error of 0.05. In both studied groups, the sample size exceeded the required number of subjects identified by power analysis.

The normality of distribution was tested using the Shapiro–Wilk W test. Normally distributed data were expressed as mean ± SD. Non-Gaussian data were presented as median (interquartile range). Comparisons between AIT and CON were performed using an unpaired t-test with Welch correction and the Mann–Whitney U test for normally and non-normally distributed variables, respectively. The χ^2^ test was used for categorical variables. Correlations among variables were assessed by Pearson’s correlations, where required, a log transformation of the variables was made for normal distribution before calculating correlations. Multiple regression analysis was performed to determine the independent influence of KP metabolites on SPINA-GD values, based on previous results of Pearson’s correlation analysis. Receiver operating characteristic (ROC) curves were prepared to evaluate the diagnostic performance of KP metabolites in AIT prediction, individually or in combination. All reported confidence interval (CI) values were calculated at the 95% level. Data were analyzed using the Statistica 13.3 software (TIBCO Software Inc., California, USA) and MedCalc software version 22.009 (MedCalc Software Ltd., Ostend, Belgium). A two-tailed *p* < 0.05 was considered statistically significant. Graphical presentation of the results was performed using GraphPad Prism 6.0 software, Boston, USA.

## Data Availability

The datasets generated during and/or analysed during the current study are available from the corresponding author upon reasonable request.
